# An ultra-high density SNP-based linkage map for enhancing the pikeperch (*Sander lucioperca*) genome assembly to chromosome-scale

**DOI:** 10.1038/s41598-020-79358-z

**Published:** 2020-12-18

**Authors:** Lidia de los Ríos-Pérez, Julien A. Nguinkal, Marieke Verleih, Alexander Rebl, Ronald M. Brunner, Jan Klosa, Nadine Schäfer, Marcus Stüeken, Tom Goldammer, Dörte Wittenburg

**Affiliations:** 1 Institute of Genetics and Biometry, Leibniz Institute for Farm Animal Biology (FBN), Wilhelm-Stahl-Allee 2, 18196 Dummerstorf, Germany; 2 Institute of Genome Biology, Leibniz Institute for Farm Animal Biology (FBN), Wilhelm-Stahl-Allee 2, 18196 Dummerstorf, Germany; 3 Mecklenburg‐Vorpommern Research Centre for Agriculture and Fisheries, Malchower Chaussee 1, 17194 Hohen Wangelin, Germany; 4 Molecular Biology and Fish Genetics, Faculty of Agriculture and Environmental Sciences, University of Rostock, 18059 Rostock, Germany

**Keywords:** Genetic linkage study, Genetic markers

## Abstract

Pikeperch (*Sander lucioperca*) is a fish species with growing economic significance in the aquaculture industry. However, successful positioning of pikeperch in large-scale aquaculture requires advances in our understanding of its genome organization. In this study, an ultra-high density linkage map for pikeperch comprising 24 linkage groups and 1,023,625 single nucleotide polymorphisms markers was constructed after genotyping whole-genome sequencing data from 11 broodstock and 363 progeny, belonging to 6 full-sib families. The sex-specific linkage maps spanned a total of 2985.16 cM in females and 2540.47 cM in males with an average inter-marker distance of 0.0030 and 0.0026 cM, respectively. The sex-averaged map spanned a total of 2725.53 cM with an average inter-marker distance of 0.0028 cM. Furthermore, the sex-averaged map was used for improving the contiguity and accuracy of the current pikeperch genome assembly. Based on 723,360 markers, 706 contigs were anchored and oriented into 24 pseudomolecules, covering a total of 896.48 Mb and accounting for 99.47% of the assembled genome size. The overall contiguity of the assembly improved with a scaffold N50 length of 41.06 Mb. Finally, an updated annotation of protein-coding genes and repetitive elements of the enhanced genome assembly is provided at NCBI.

## Introduction

Pikeperch (*Sander lucioperca)* is a freshwater fish species from the Percidae family native to Europe and Asia^1,2^. Its meat quality, with low fat content and high protein^3^, has placed it as a fish of high commercial value and a candidate for intensive inland aquaculture. In a period of 10 years, from 2007 to 2017, the global capture production of pikeperch increased from 17,891 to 20,481 tonnes, while the global inland aquaculture production increased from 627 to 1418 tonnes^4^, making evident the growing demand for this species.

Several studies have been performed in pikeperch concerning productive (e.g., growth and survival)^5–7^ and reproductive (e.g., fecundity and spawning)^8,9^ traits. However, despite the growing commercial importance of this species, little information is available regarding its genetic and genomic makeup. In 2018, the first high-density linkage map of pikeperch was built using specific locus amplified fragment sequencing (SLAF-seq). The map consisted of 8159 SLAFs including 8767 single nucleotide polymorphisms (SNPs) markers in 24 linkage groups (LGs) and spanned 3421.81 cM, with an average inter-marker distance of 0.46 cM^10^.

Linkage analysis of high-density genomic markers has facilitated the assembly of reference genomes by anchoring scaffolds, produced during de novo genome assembly, into linkage groups and providing a chromosome frame^11^. The resulting linkage maps provide useful information or even the essential basis for the analysis of sex-related structural differences and inheritance patterns^12^. Furthermore, linkage maps are often used for the detection of chromosomal locations of functional or disease genes and the identification of quantitative trait loci (QTLs) associated to economically important traits^13,14^. Several linkage maps have been produced for a number of fish species and used with different purposes. In common carp (*Cyprinus carpio*) and yellow drum (*Nibea albiflora*), high-density linkage maps were built for comparative genomic analysis and identification of QTLs for growth and sex related traits^15,16^. A linkage map produced in European whitefish (*Coregonus* sp. “Albock”) helped to investigate its genomic basis of adaptation and speciation^17^. Recently, in channel catfish (*Ictalurus punctatus*), a high-density linkage map was used for the construction of chromosome maps^18^.

With the fast advancements in next-generation sequencing technologies, an increasing number of sequencing and genotyping methodologies for SNPs have been developed, making it possible to rapidly discover a huge number of markers at relatively low cost^19,20^. The challenge persists to arrange this excessive amount of genetic information into physical coordinates. The first highly contiguous draft genome assembly of pikeperch was published recently^21^. It contained ~ 900 Mb of total sequence, comprising 1966 contigs ordered into 1313 scaffolds. However, this first draft assembly is fragmented and requires improvement to a chromosome-scale. Genomes with accurate and complete architecture provide additional genomic context by orienting genes relative to each other and helping to determine other genomic features such as centromeres, telomeres, complex repeat elements and regulatory regions^22^. Assemblies with low integrity and completeness have been one of the major limitations to improve research in aquaculture species^23,24^. Therefore, a linkage analysis is urgently required to build a basis for upgrading the current pikeperch genome, and developing breeding strategies in pikeperch aquaculture.

In this study, we report the construction of an ultra-high density linkage map for pikeperch based on the most common form of genetic variation, i.e., SNPs, and the improvement of the pikeperch genome assembly to chromosome-scale. The workflow described covers tissue sampling to raw sequence data, to finally yield a large panel of hard-filtered SNPs. A linkage map was constructed using the software Lep-Map3^25^ suited to sequence data and capable of handling millions of markers, and the characteristics of the 24 resulting linkage groups are reported. The generated linkage map was then used to enhance the pikeperch genome assembly by anchoring and ordering its scaffolds into chromosome-scale pseudomolecules. The key genomic features were annotated for the enhanced pikeperch genome, including coding genes, non-coding RNA, and various repeat elements.

## Results

### Sequence processing and genotyping

**Figure 1:**
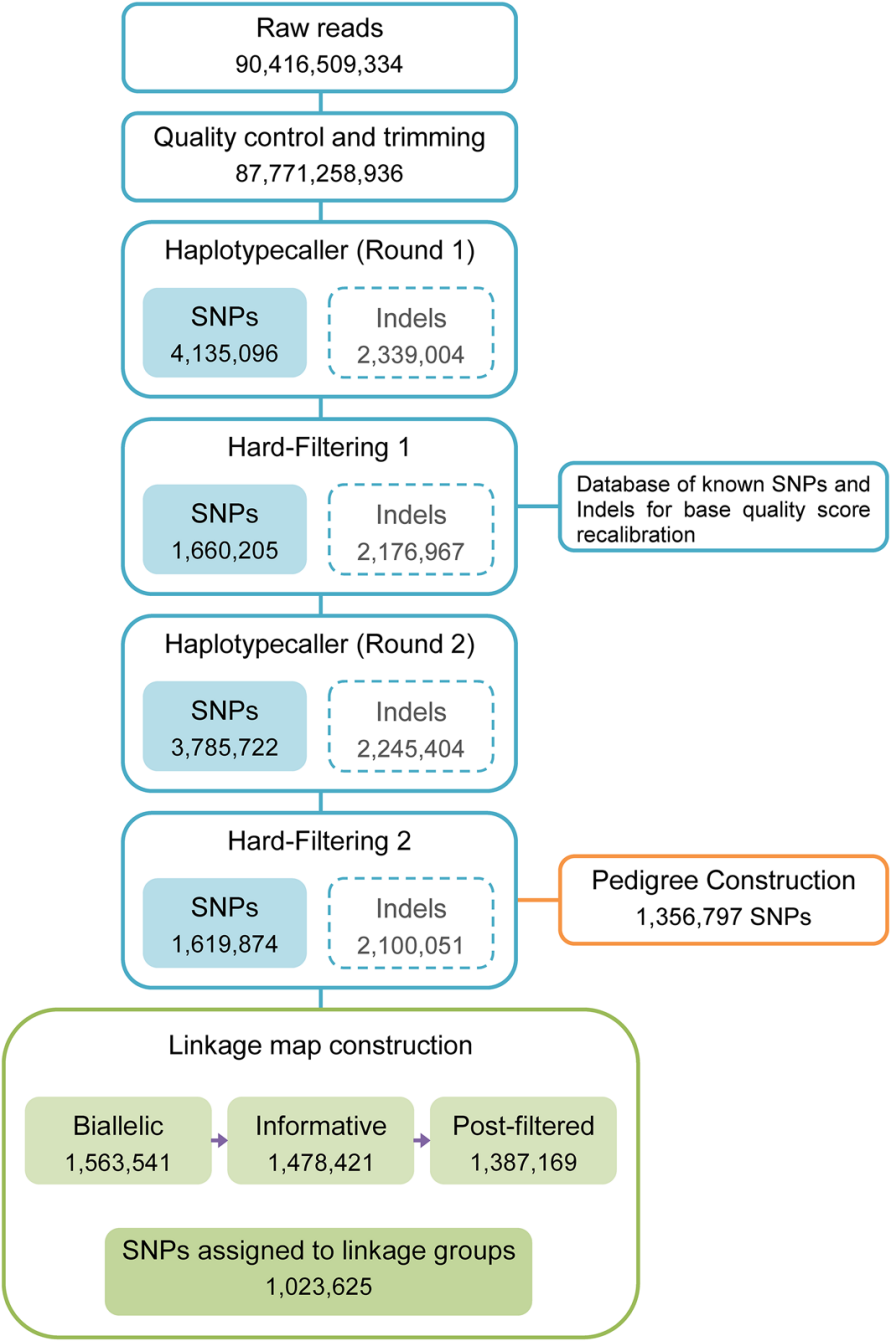
Pipeline showing the number of variants involved in the different steps. SNPs hard-filtering criteria: QualByDepth (QD) < 10.0, Quality (QUAL) < 30.0, StrandOddsRatio (SOR) > 3.0, FisherStrand (FS) > 60.0, RMSMappingQuality (MQ) < 40.0, MappingQualityRankSumTest (MQRankSum) < -12.5 and ReadPosRankSumTest (ReadPosRankSum) < -8.0. Indels hard-filtering criteria: QualByDepth (QD) < 2.0, Quality (QUAL) < 30.0, FisherStrand (FS) > 200.0 and ReadPosRankSumTest (ReadPosRankSum) < -20.0.

A total of 90,416,509,334 paired-end reads (151 bp) from the 394 pikeperch samples were generated with an average number of 229,483,526 reads per sample and an average of 31.08-fold coverage. After trimming and quality filtering, a total of 87,771,258,936 paired-end reads were retained, with an average number of 222,769,693 reads per sample. The average percentage of properly paired reads was 96.43%. Although the Genome Analysis Toolkit v4.0 (GATK) variant calling pipeline^26^ simultaneously discovers SNPs and Indels, we focused only on the SNPs and obtained a total of 1,619,874 SNPs after hard-filtering. For completeness, results for both types of variants are shown in Fig. 1.

### Pedigree construction

Results from the pedigree showed that the 375 pikeperch sampled from the pool of progeny belonged to six out of the seven matings performed at the fish facility. Four of the families were full-sibs and two other full-sib families built one paternal half-sib family. The number of progeny corresponding to each mating is shown in Table 1. The mating, from which no progeny was found, was reported to have a very low number of eggs. Additionally, two more matings had extremely few progeny, which could be related to multiple factors, such as fertilization and hatching rate^27,28^, stocking density^29^, size sorting^30^, and cannibalistic behaviour in early stages^31^, among others.

**Table 1:** Matings and number of individuals sampled from each family.

Family	Sire Id	Dam Id	Number of progeny
1	1	2	29
2	3	4	98
3	5	6	3
4	7	8	224
5	9	10	15
6	9	11	6

### Linkage map construction

**Figure 2:**
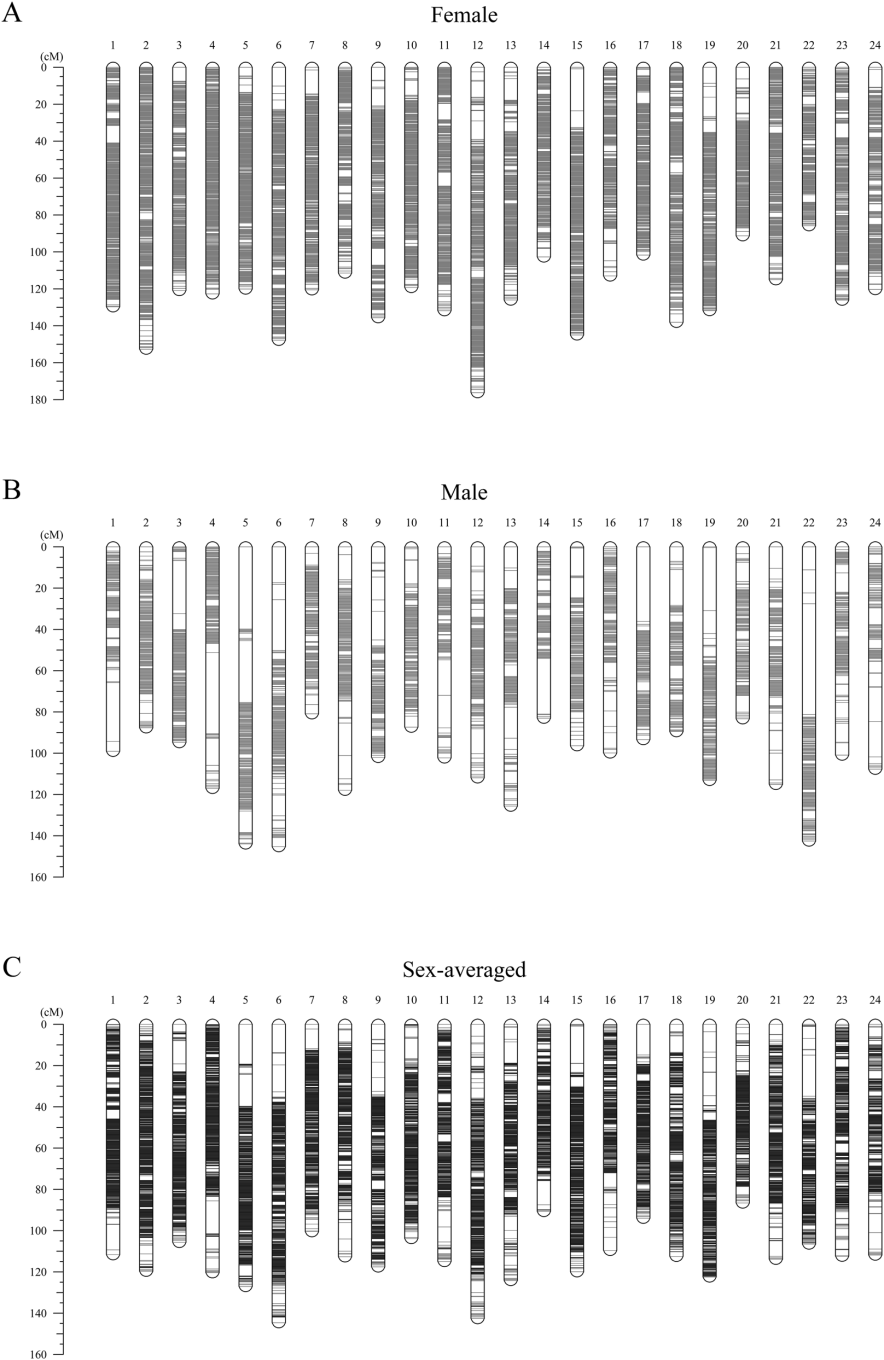
Genetic positions of markers for the 24 linkage groups in the (**a**) female, (**b**) male and (**c**) sex-averaged linkage maps. A black bar represents a SNP marker. The scale on the left indicates the genetic position in centiMorgan (cM).

From the 1,563,541 initial biallelic variants, 1,478,421 were identified as informative out of which 91,252 were discarded after filtering by segregation distortion and after allowing at most 10% of missing genotypes. Hence, a total of 1,387,169 variants were kept for further analysis. A range of logarithm of odds (LOD) scores from 5 to 70 incrementing by 5 was tested for linkage grouping. A LOD score of 50 resulted in 24 LGs that were expected to match to the 24 chromosomes observed in karyotype studies in pikeperch^32,33^. In total, 1,023,625 SNPs were uniquely assigned to the 24 LGs and ordered to generate the female, male and sex-averaged linkage maps (Table 2, Fig. 2). The number of SNPs per LG ranged from 28,022 to 59,051 with an average of 42,651 markers per LG. In total, 863 out of 1313 scaffolds were involved, covering 894.02 Mb of the total genome length of 900.48 Mb. The number of SNPs per scaffold ranged from 1 to 25,495 with mean 1186. Out of the 863 scaffolds, 65 had only one SNP and 15 had more than 10,000 SNPs, while all magnitudes in between were almost equally represented: 136 scaffolds contained two to 10 SNPs, 165 scaffolds included 11 to 100 SNPs and 1001 to 10,000 SNPs were found in 209 scaffolds.

**Table 2:** Description of the female, male and sex-averaged linkage maps. LG: linkage group, cM: centiMorgan, F:M: female:male.

LG	Number of SNPs	Female map				Male map				Sex-averaged map				F:M length ratio
		Distinct positions	LG Length (cM)	Average inter-marker distance (cM)	Max gap (cM)	Distinct positions	LG Length (cM)	Average inter-marker distance (cM)	Max gap (cM)	Distinct positions	LG Length (cM)	Average inter-marker distance (cM)	Max gap (cM)	
1	59,051	381	129.69	0.0022	9.37	135	99.05	0.0017	28.58	508	111.65	0.0019	12.28	1.31
2	57,505	441	152.63	0.0027	3.72	197	87.68	0.0015	5.53	626	119.66	0.0021	4.16	1.74
3	51,714	325	120.87	0.0023	7.48	187	94.85	0.0018	25.73	505	105.69	0.0020	11.23	1.27
4	53,468	392	122.71	0.0023	2.44	163	117.02	0.0022	39.42	550	120.40	0.0023	15.92	1.05
5	49,759	345	120.06	0.0024	4.69	184	143.95	0.0029	39.82	518	127.05	0.0026	19.23	0.83
6	56,492	380	147.84	0.0026	10.12	192	145.25	0.0026	24.69	557	144.61	0.0026	13.59	1.02
7	43,973	336	120.50	0.0027	12.98	174	80.88	0.0018	5.74	501	100.51	0.0023	9.48	1.49
8	43,504	272	111.41	0.0026	4.01	188	117.91	0.0027	15.77	449	112.68	0.0026	8.00	0.94
9	36,159	313	135.60	0.0037	9.37	148	102.00	0.0028	13.88	444	117.58	0.0033	7.41	1.33
10	49,364	337	119.22	0.0024	8.08	175	87.34	0.0018	11.35	504	103.80	0.0021	9.69	1.37
11	45,511	340	131.79	0.0029	8.71	140	102.26	0.0022	17.27	473	114.95	0.0025	7.89	1.29
12	52,318	405	176.19	0.0034	10.03	177	111.91	0.0021	12.06	564	142.63	0.0027	5.77	1.57
13	42,566	278	126.05	0.0030	11.75	185	125.63	0.0030	14.99	443	124.13	0.0029	10.20	1.00
14	38,321	285	102.72	0.0027	4.55	120	83.00	0.0022	26.95	398	90.70	0.0024	11.68	1.24
15	42,062	370	144.93	0.0034	22.77	184	96.34	0.0023	13.52	548	119.93	0.0029	17.93	1.50
16	35,329	257	113.01	0.0032	9.37	161	99.85	0.0028	9.37	401	109.55	0.0031	6.93	1.13
17	35,432	298	101.64	0.0029	6.15	143	93.37	0.0026	36.11	436	93.99	0.0027	14.86	1.09
18	35,434	362	138.10	0.0039	5.84	146	89.53	0.0025	17.27	490	112.45	0.0032	7.89	1.54
19	33,516	316	131.74	0.0039	10.37	182	113.13	0.0034	30.61	483	122.43	0.0037	11.27	1.16
20	28,022	223	91.21	0.0033	8.05	148	83.28	0.0030	9.36	365	86.59	0.0031	4.77	1.10
21	30,845	330	115.03	0.0037	3.72	157	115.09	0.0037	12.98	476	113.86	0.0037	7.49	1.00
22	29,046	226	85.79	0.0030	4.31	168	142.53	0.0049	53.73	386	106.49	0.0037	19.97	0.60
23	42,635	328	126.10	0.0030	7.73	152	100.89	0.0024	9.37	464	112.37	0.0026	4.56	1.25
24	31,599	265	120.35	0.0038	9.60	111	107.74	0.0034	17.27	370	111.83	0.0035	9.17	1.12
Total	1,023,625	7,805	2,985.16	–	–	3,917	2,540.47	–	–	11,459	2,725.53	–	–	–
Average	42,651	325.21	124.38	0.0030	8.13	163.21	105.85	0.0026	20.47	477.46	113.56	0.0028	10.47	1.21

The SNPs on the female map were arranged on 7805 distinct positions with observed recombination events constituting a total genetic length of 2985.16 cM. The genetic length of LGs ranged from 85.79 cM (LG22) to 176.19 cM (LG12) with an average length of 124.38 cM. The average inter-marker distance was 0.0030 cM with the smallest and largest distance being 0.0022 (LG1) and 0.0039 (LG18 and LG19). The largest gap between adjacent markers was of 22.77 cM (LG15).

The SNPs on the male map were arranged on 3917 distinct positions with observed recombination events constituting a total genetic length of 2540.47 cM. The genetic length of LGs ranged from 80.88 cM (LG7) to 145.25 cM (LG6) with an average length of 105.85 cM. The average inter-marker distance was 0.0026 cM with the smallest and largest distance being 0.0015 cM (LG2) and 0.0049 cM (LG22). The largest gap between adjacent markers was of 53.73 cM (LG22). The female:male (F:M) length ratio for the LGs varied from 0.60 (LG22) to 1.74 (LG2), with an average of 1.21. The LGs showed different recombination activities between the female and male maps; Figure S1 shows its non-linear relationship. Furthermore, 18 LGs showed larger genetic distances in females than in males. In contrast, three LGs (LG5, LG8 and LG22) showed larger genetic distances in males than females. Three LGs (LG6, LG13 and LG21) had approximately the same length between sexes.

The SNPs on the sex-averaged map were arranged on 11,459 distinct positions with a total genetic length of 2725.53 cM. The genetic length for the LGs ranged from 86.59 cM (LG20) to 144.61 cM (LG6) with an average length of 113.56 cM. The average inter-marker distance was 0.0028 cM with the smallest and largest distance being 0.0019 (LG1) and 0.0037 (LG19, LG21 and LG22). The largest gap between adjacent markers was 19.97 cM (LG22).

### Genome assembly and annotations

The generated de novo assembly consisted of 1602 contigs with N50 size of 6.3 Mb, which is more than a twofold improvement over the previously published draft assembly (GenBank accession: PRJNA561467). The integrated chromosome-scale assembly yielded 336 scaffolds with N50 size of 41.06 Mb from which the 24 largest scaffolds represented the putative 24 pikeperch chromosomes, and covered 896.48 Mb (99.47%) of the assembly size. Only 4.74 Mb (0.53%) could not be anchored into pseudomolecules. The average accuracy at base-level was 99.9996 (i.e., 1 error in 100 kb). Over 99.80% of the genomic paired-end reads mapped to the improved assembly, with 97.50% of them mapping concordantly. Moreover, from a total of 4584 actinopterygians core genes, BUSCO assessment recovered 94.50% as full-length single-copy, 2.23% as duplicated, 1.59% as fragmented and 1.68% were missing, indicating that most genes were accurately assembled (Table 3).

**Table 3:** Comparison of statistics between our chromosome-scale assembly and the first published pikeperch draft assembly (GenBank accession PRJNA561467). Genome annotation metrics were taken from Nguinkal et al. (2019)^21^. Differences between the statistic results shown in this table and NCBI are due to the use of different genome annotation services.

	Chromosome-scale assembly	Draft assembly
**Assembly metrics**		
Total assembly size (bp)	901,221,791	900,477,756
Number of contigs	1048	1966
Contig N50 length (bp)	6,348,792	2,995,800
Number of scaffolds	336	1313
Scaffold N50 length (bp)	41,060,379	4,929,547
Longest scaffold size (bp)	54,393,628	19,065,786
Scaffold L50	10	52
Base-level accuracy	99.9996 (Q50)	99.998 (Q40)
Σ Scaffolds > 10 Mb (% of assembly size)	99.47	26.60
Σ Unplaced scaffolds (% of assembly size)	0.53	-
GC-content (%)	41.00	40.91
**Assembly completeness (Actinopterygii dataset)**		
Complete BUSCO	4434 (96.73%)	4413 (96.27%)
Complete and single copy BUSCO	4332 (94.50%)	4301 (93.83%)
Complete and duplicated BUSCO	102 (2.23%)	112 (2.44%)
Fragmented BUSCO	73 (1.59%)	89 (1.94%)
Missing BUSCO	77 (1.68%)	82 (1.79%)
**Genes annotation**		
Number of genes	36,010	24,278
Number of protein-coding genes	33,456	21,249
Mean gene length (bp)	10,697	10,961
Mean CDS length (bp)	1451	1313
Mean exon count per CDS	7.80	6.70
Coding genes with homology-based functional annotation	31,234 (93.36%)	18,536 (87.23%)
Mean intron length (bp)	2276	1696
Mean exon length (bp)	156	196
% of genome covered by exons	3.82	3.11
Number of tRNA	2345	2313
Numer of rRNA	160	180
Number of miRNA	145	166

Homology and structure-based approaches were used for functional annotation of protein-coding genes. We found 31,234 genes (93.36% of protein-coding genes) with at least one significant hit in one of the functional databases queried. The predicted non-coding genes included 2345 transfer RNA (tRNA), 160 ribosomal RNA (rRNA) and 145 microRNA (miRNA) (Table 3).

**Figure 3:**
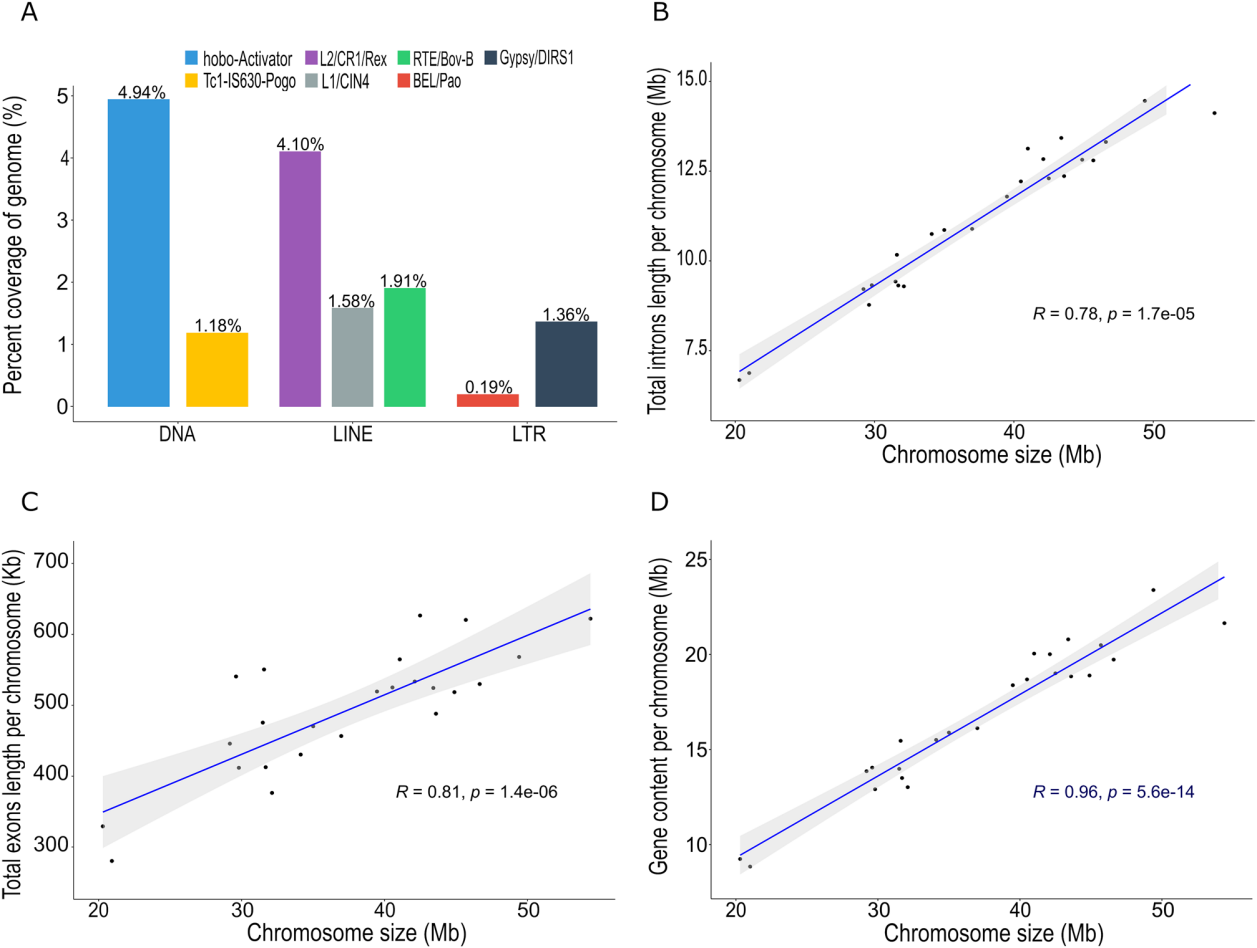
(**a**) The percentage coverage of the most abundant families of transposable elements in pikeperch. LINE: long interspersed nuclear elements; LTR: long terminal repeat. Correlation between (**b**) total introns length, (**c**) total exons length, and (**d**) gene content per chromosome and the pikeperch chromosome size (Mb).

Repetitive sequences accounted for ~ 37% of the assembled genome, and spanned 334 Mb in total, which is in range with the repeats content reported in other Percidae fish^34^. With more than 250 Mb (27.76% of assembly size), DNA transposons and retroelements were the most abundant type of repeats found in the pikeperch genome. In particular, long interspersed nuclear elements (LINEs), long terminal repeat (LTR) elements and hobo-Activator occupied 10.16%, 3.22% and 4.94%, respectively, of the assembled genome (Fig. 3a).

**Figure 4:**
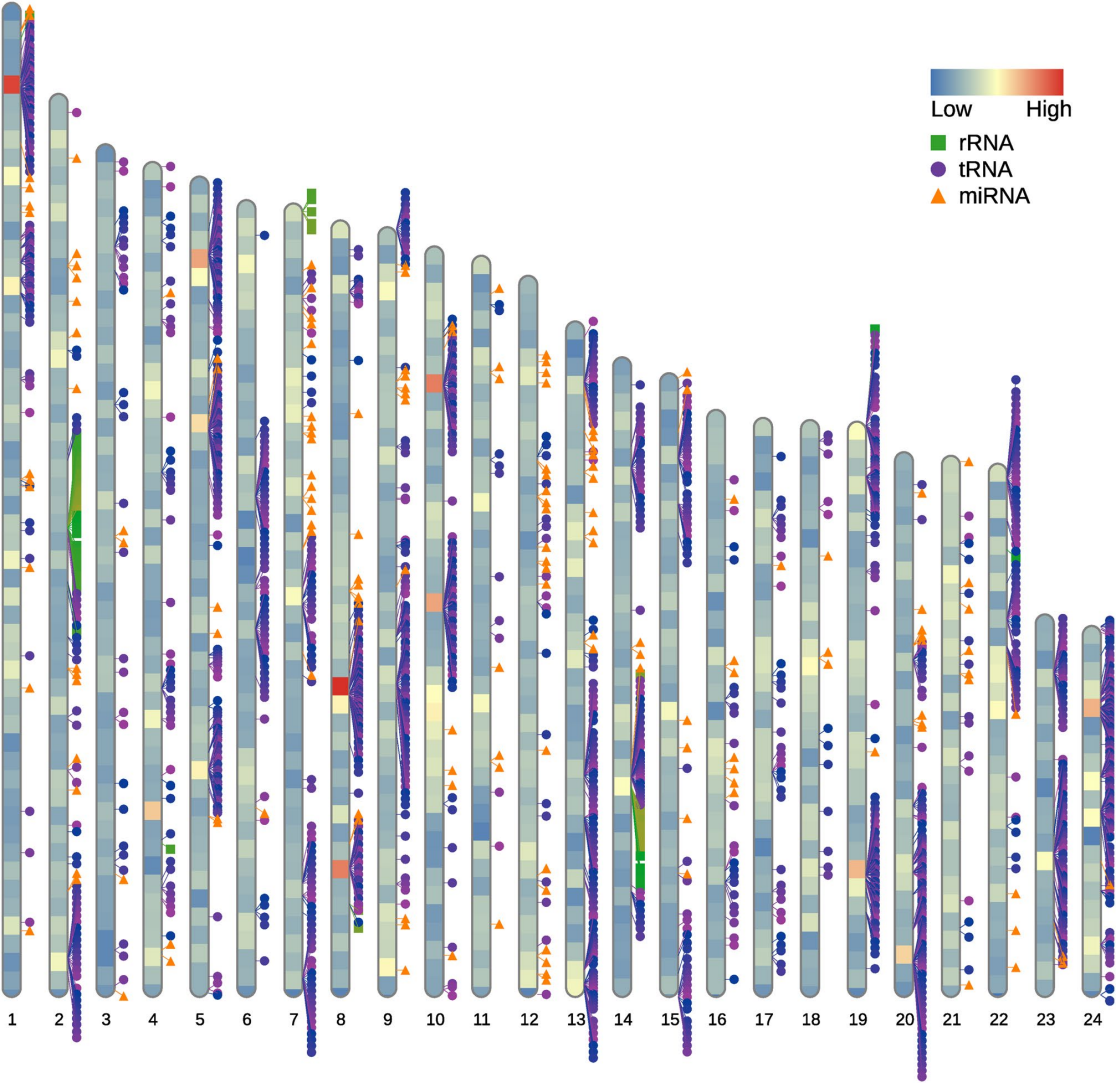
Gene density on each pikeperch chromosome ordered by length and distribution of non-coding RNA loci including miRNA (orange triangle), tRNA (purple circle) and rRNA (green square). The colour code within each chromosome represents the gene density from low (blue) to high (red) in a window of 1 Mb.

The obtained consensus gene models included a total of 33,456 high-quality protein-coding genes, which was substantially higher than that found in the previously published draft assembly (GenBank accession: PRJNA561467) version. The average length of coding sequences (CDS) was 1451 bp. On average, each *S. lucioperca* gene had 7.8 exons, each with an average length of 156 bp. About 82% of the 278,346 exonic sequences were < 200 bp. Introns showed an average length of 2276 bp, with 2% of them having a length of > 10 kb. Moreover, the total length of intronic and exonic DNA on each chromosome was significantly correlated to the chromosome size with correlation coefficients of R = 0.78 and R = 0.81, respectively (Fig. 3b,c). Consequently, the gene content per chromosome was also significantly correlated to the chromosome size, with a correlation coefficient of R = 0.96 (Fig. 3d). Overall, the distribution of CDS length, intron length and exon number is comparable with other percid genomes^34^. The 24 chromosomes were sorted by physical size, from largest to smallest and named accordingly (Table 4, Fig. 4). Given a genome-wide average of 40 genes per Mb, the chromosomes 21 and 23 displayed the highest and lowest gene density with 52 and 34 genes per Mb, respectively. Additionally, we observed a putative nucleolus organizer region (NOR) on chromosome 7, which had already been observed in previous cytogenetics analysis on pikeperch^35^.

**Table 4:** Description of chromosomes ordered by size with corresponding LG. LG: linkage group, Mb: Megabase.

Chromosome	LG	No. of anchored markers	Integrated contigs	No. of genes	Physical length (Mb)	Gene density (genes/Mb)
1	15	33,239	33	2095	54.39	38.52
2	4	38,017	26	2071	49.41	41.92
3	1	38,493	34	1598	46.65	34.25
4	2	40,980	24	1846	45.68	40.41
5	6	33,387	48	1675	44.88	37.32
6	12	35,005	31	1722	43.59	39.50
7	3	41,147	24	1692	43.41	38.98
8	10	29,918	35	1739	42.48	40.94
9	5	40,404	33	1793	42.11	42.58
10	23	27,205	25	1777	41.06	43.28
11	11	32,750	29	1668	40.55	41.14
12	18	24,823	41	1697	39.47	43.00
13	19	28,731	24	1329	36.97	35.95
14	9	24,299	29	1260	35.01	35.99
15	7	28,542	24	1249	34.13	36.59
16	24	18,151	38	1263	32.13	39.31
17	13	28,606	58	1288	31.68	40.65
18	17	33,245	27	1383	31.57	43.81
19	21	23,452	26	1288	31.48	40.91
20	22	21,440	14	1202	29.81	40.32
21	8	31,401	19	1531	29.61	51.70
22	14	25,569	31	1254	29.18	42.98
23	16	20,259	19	708	20.93	33.83
24	20	24,297	14	864	20.30	42.57
Total	-	723,360	706	35,992	896.48	-
Average	-	30,140	29.42	1500	37.35	40.27

A liftover of the SNPs assigned to LGs to the chromosome-scale build yielded a panel of 992,340 genome-wide reference SNPs for pikeperch (Table S2). In total, 31,278 SNPs failed to map to the chromosome-scale assembly and 7 duplicated SNPs were removed.

## Discussion

We reported the construction of an ultra-high density SNP-based linkage map for pikeperch, and the further anchoring of the genome assembly into the first chromosome-scale assembly.

Our map comprised 24 linkage groups, with a total of one million SNP markers which spanned between 2500 and 3000 cM for the female, male and sex-averaged maps. In order to obtain a high quality linkage map, we strictly filtered the data and finally retained 1.6 million SNP markers from sequence data. Roughly, 600 K SNPs could not be assigned to any LG. The female map was slightly longer than the male map, with an overall F:M length ratio of 1.21, though some LGs harboured extreme differences between genders (F:M length ratio up to 1.74). This result was consistent with the only linkage map reported in pikeperch, where the female map was also found to be longer than the male map, with an overall F:M length ratio of 1.62 (4179.41 cM vs. 2582.83 cM)^10^. Our results are also consistent with the pattern between sexes in several teleost fish species like red-spotted grouper (1.47 F:M length ratio^36^), Pacific bluefin tuna (1.34 F:M length ratio^37^) and barramundi (2.1 F:M length ratio^38^).

Average inter-marker distances were between 0.0026 cM and 0.0030 cM for the female, male and sex-averaged map leading to a more than 100 times higher resolution compared with the linkage map published by Guo et al.^10^. Additionally, our linkage map was based on about one million SNPs from whole-genome sequencing of 6 full-sib families comprising 363 progeny, while the map derived by Guo et al. was built using 8767 SNPs from a single family with 150 progeny. Though the total length of male map was almost equal, the female map length differed being 1.4 times longer in the earlier study. However, a larger mapping population, enormously increased number of markers, and thus essentially smaller average inter-marker distance, substantiated a more precise estimation of the genetic distances. Because of the close proximity of SNPs, recombination events rarely happened within scaffolds and this was manifested by genetic positions hardly differing within long stretches, see Supplementary Table S1. Though being beneficial at the large scale, ordering of markers at the fine scale might be insufficient based on linkage analysis only^11^. However, high-quality linkage maps are a valuable source for the correct placement of scaffolds into chromosomes^39^. Our ultra-high density linkage map was used to anchor the genome scaffolds into chromosome-scale. Compared to the previous genome assembly^21^, the scaffold N50 length was increased from 4.9 Mb to 41.06 Mb covering 896.48 Mb (99.47%) of the assembly size. This new chromosome-scale genome assembly represents an important resource to fill the gap in the Percidae family tree, where Luciopercinae (*Sander* spp.) was the only sub-family missing a chromosome-level assembly (according to NCBI query: June 2020).

Anchoring scaffolds into chromosomes has been performed with Chromonomer software (http://catchenlab.life.illinois.edu/chromonomer/). Alternatively, well-suited software such as Lep-Anchor^40^ or ALLMAPS^41^ provide potential for further advances.

The conversion of the genomic positions of SNPs into the chromosome-scale assembly successfully lifted over 96.94% of the markers. The remaining 3.06% (31,278) of the SNPs could not be lifted over because they resided in contigs that only existed in the older assembly build or because of sequence incompatibilities between the assemblies, such as mismatching reference alleles, e.g., a variant that was considered an alternate in the source assembly was now considered the reference in the target assembly. Additionally, 7 SNPs were found to be duplicated; they mapped to the same physical position because of collapsing or overlapping contigs in the target assembly.

The karyotype of the pikeperch consists of one pair of metacentric, 15 pairs of submetacentric and 8 pairs of subtelo-acrocentric chromosomes^32,33^. The number of linkage groups for the female, male and sex-averaged maps built in this study was chosen corresponding to the number of chromosome pairs from microscopic observations^32,33^. With the aim of identifying the chromosome type and specifying the location of centromeric regions, we applied the centromere mapping method developed by Limborg et al*.* (2015)^42^ to all linkage groups of our female map. This required recombination frequencies (RF) between each of the two terminal markers and any other marker (m) on each linkage group. The resulting RF_m_ curves shall indicate a metacentric chromosome if the two curves cross at almost 0.5 and an acrocentric chromosome if the curves smoothly approach 0.5 at the ends. In our study, this method did not allow for a clear differentiation between metacentric and acrocentric LGs and therefore, remained inconclusive (Supplementary Figure S2). In order to account for possible genotype errors at the terminal markers, markers close to them have been verified, and they confirmed the inconclusive outcome. As mentioned by Limborg et al. (2015)^42^, this method has reduced precision if recombination interference is incomplete and chromosome arms are long (> 50 cM). This leads to an increasing frequency of double crossover events inducing RF_m_ to level off after ~ 50 cM. This was observed in our study, possibly indicating incomplete interference in pikeperch. Thus, further research is needed to elucidate the extent of interference and to narrow down the location of the centromere by studying regions with repressed recombination activity^43^. Once centromeres have been identified, the order of chromosomes will change accordingly.

The development of genomic resources for pikeperch will allow a better understanding of the species and a faster positioning in the aquaculture industry. Pushed by the advancements in high-throughput methods for SNP genotyping, genomic selection has been introduced in some aquaculture breeding programs, but further research is needed to effectively combine existing breeding designs with available genomic information^44^. Mapping of genomic regions associated with diseases will provide further possibilities to accelerate the breeding success in aquaculture species^45^. Moreover, the genomic resources generated in this project will serve for various future studies, including the improvement in the contiguity and accuracy of the chromosome-scale assembly for pikeperch and the development of a SNP array.

## Material and methods

All procedures involving the handling and treatment of fish used in this study were approved by the Committee on the Ethics of Animal Experiments of Mecklenburg-Western Pomerania (Landesamt für Gesundheit und Soziales LAGuS). Approval ID: 7221.3–1-009/19. The methods were performed in accordance with relevant guidelines and regulations.

### Broodstock management and family production

Seven matings of pikeperch were generated in a state´s aquaculture facility in Hohen Wangelin (State Research Institute for Agriculture and Fisheries in Hohen Wangelin, Mecklenburg-Western Pomerania, Germany) within their normal production cycle. For the production of the families, mature broodstock were placed in spawning tanks using a sex ratio of 2:1 and 1:1. Spawning tanks dimensions were 1.17 × 0.88 × 1.10 m (l × w × h) with a water column of 1.0 m kept at 12 °C and daily water exchange rate of 5%. Broodstock were fed with a diet for trout broodstock containing 44% protein. After spawning, per family eggs were collected and treated to prevent bacterial and fungal growth. The treatment consisted of a 10 min bath in a solution made of 50 ml of 37% formalin in 10 L of water. Eggs were then placed in incubation tanks in a small scaled recirculating aquaculture system (RAS) with continuous aeration, cooling system and UV-disinfection. After a 24 h hatching period, all obtained progeny from each family were mixed and transferred into round tanks each with a water column of 0.5 m and kept at a water temperature of 15 °C and daily water exchange rate of 5%. As first exogenous prey, larvae were fed with marine copepods in the first two days, followed by *Artemia* spp*.* for the next 10 days, and were then adapted to dry food. After 45 days with a mean weight of 0.5 g, family mixed larvae were stocked in round tanks with a water volume of 3 m^3^ at a water temperature of 21 °C and daily water exchange rate of 5%. Larvae were fed with dry food containing between 50 to 64% protein according to the growth stage; daily diet consisted of 5% to 10% of the biomass of the tank.

### DNA extraction and sequencing

Genomic DNA from the 18 broodstock (11 males and 7 females) used for the family production was isolated from flash-frozen caudal fin tissue sampled after mating. One male fish was used twice, giving a total of 19 samples from 18 different individuals. A total of 375 progeny were collected for sampling at the age of 16 and 28 weeks. Genomic DNA from the progeny was isolated from blood obtained from the caudal vein or flash-frozen caudal fin. For both, broodstock and progeny, genomic DNA isolation was performed using DNeasy Blood and Tissue Kit (Qiagen) and following manufacturer’s protocol. DNA quantity and quality were determined with the NanoDrop ND-1000 spectrophotometer (NanoDropTechnologies, Wilmington, Delaware, USA). Whole genome paired-end sequencing was performed on each of the individuals (Macrogen, Korea) with Illumina NovaSeq 6000 technology.

### Sequence processing and genotyping

The pikeperch draft assembly (GenBank accession: PRJNA561467)^21^ was used as reference genome. This draft assembly consists of ~ 900 Mb of total sequence, comprising 1966 contigs ordered into 1313 scaffolds with N50 lengths of 3.0 Mb and 4.9 Mb, respectively. In total, 394 whole genome paired-end sequences were genotyped. Quality control of sequencing data was performed with FastQC v0.11.7^46^. Fastp v0.19.10^47^ was used for adapter and overrepresented sequences trimming. Short variants (SNPs and Indels) were discovered following the Genome Analysis Toolkit v4.0 (GATK) pipeline^26^: (i) Burrows-Wheeler Aligner (BWA-MEM)^48,49^ was used to map the reads of each sample to the reference genome. (ii) Picard tools were used to sort the SAM files and mark duplicates. (iii) Variants were called using the HaplotypeCaller tool. Since no database of known SNPs and Indels was available for pikeperch, such a database was bootstrapped. First, an initial round of variant calling was performed. Then, SNPs and Indels with the highest confidence were used as database of known SNPs and Indels and fed into the base quality score recalibrator. Details on how to bootstrap a set of known variants can be found at https://gatk.broadinstitute.org/hc/en-us/articles/360035890531-Base-Quality-Score-Recalibration-BQSR-. Finally, a second round of variant calling with the recalibrated data was performed. SNPs were hard-filtered by the following criteria: QualByDepth (QD) < 10.0, Quality (QUAL) < 30.0, StrandOddsRatio (SOR) > 3.0, FisherStrand (FS) > 60.0, RMSMappingQuality (MQ) < 40.0, MappingQualityRankSumTest (MQRankSum) < -12.5 and ReadPosRankSumTest (ReadPosRankSum) < -8.0. Details on how to choose the filter criteria can be found at https://gatk.broadinstitute.org/hc/en-us/articles/360035890471-Hard-filtering-germline-short-variants.

### Pedigree construction

The production procedures at the fish facility did not allow for the identification of the successful male in 2:1 matings, as well as the progeny belonging to each mating. Therefore, the reconstruction of a pedigree was required. This was carried out using the parentage assignment algorithm AlphaAssign^50^. Prior to this, the VCF file containing the set of SNPs, which remained after hard-filtering, was recoded with PLINK 1.9^51^ according to AlphaAssign requirements. Additionally, we kept only the bi-allelic variants without missing genotypes. Then 18 putative parents and 375 progeny with 1,356,797 markers were included in the analysis. Due to the fact that the sampled individuals came from an inbred population, additional filtering for Hardy–Weinberg equilibrium and minor allele frequency were not considered.

### Linkage map construction

Building of LGs and ordering of SNPs within LGs were performed using Lep-Map3^25^. Prior to this, the VCF file containing the SNPs after hard-filtering was processed with the GATK tool SelectVariants to keep only the bi-allelic markers and to remove the samples of individuals from the broodstock that did not have progeny. Additionally, BCFtools was used to check for Mendelian errors, and after visual inspection, individuals with > 2% Mendelian error rate were discarded. A total of 11 broodstock and 363 progeny, from 6 full-sib families, and 1,563,541 markers were included in the analysis. Lep-Map3 modules were used starting with the ParentCall2 module to identify informative markers. Then, the Filtering2 module was used to remove markers with segregation distortion (dataTolerance = 0.01) and missing genotypes > 10% (MissingLimit = 0.1). The assignment of markers into LGs was performed with the SeparateChromosomes2 module with a LOD score of > 50 (LodLimit = 50). The JoinSingles2All module was used for assigning singular markers to existing LGs with a LOD score of > 30 (LodLimit = 30) and a LOD difference of > 10 (lodDifference = 10). Markers that could not be uniquely assigned to any LG were discarded. The OrderMarkers2 module was used to order the markers within each LG. The output of this module consisted of marker names with paternal and maternal map position in centiMorgan units. The ordering of markers within this module was based on random number generation. To account for the occurring stochastic variation, this step was repeated 10 times for each LG. For each LG the ordering of markers with highest haplotype likelihood was taken to build the final map. Sex-specific genetic positions were reported. The sex-averaged genetic positions were obtained via the module OrderMarkers2 (sexAveraged = 1).

### Genome assembly, scaffolds anchoring and reference SNPs set

The pikeperch draft assembly (GenBank accession: PRJNA561467) was upgraded in two steps. First, we generated a new de novo assembly with the previously released sequencing data of *S. lucioperca*^21^*,* i.e. the genomic PacBio Single-molecule real-time sequencing (SMRT) reads (Accession: SRX6760932) and Illumina paired-end data (Accession: SRX6750544) available at NCBI BioProject PRJNA561467. For a comprehensive description of these data, please refer to Nguinkal et al. (2019)^21^. The MaSuRCA Genome Assembly and Analysis Toolkit v.3.4.02^52^ was used to construct this de novo assembly.

Second, flanking sequences of the SNP loci (100 bp upstream and 100 bp downstream from the SNP) of the sex-average genetic map were extracted and aligned to the de novo assembled genome with BWA^48^. In total, 723,360 markers uniquely mapped to 706 contigs which were ordered and integrated into 24 pseudomolecules using the software Chromonomer v1.11 (http://catchenlab.life.illinois.edu/chromonomer/). The genome was polished in three iterations with POLCA^52^ using short paired-end reads. The structural accuracy of the anchored assembly was assessed by mapping the 394 whole-genome paired-end sequences generated in this study (BioProject: PRJNA626522) to the genome, and the gene space completeness was measured by analysing the rate of highly conserved single-copy orthologs in the ray-finned fish lineage, using BUSCO v3 tools^53^.

In order to obtain the final reference SNP panel, all SNPs assigned to LGs were lifted over to this polished and gap-filled chromosome-scale build: (i) A chain file was produced with flo^54^. (ii) Crossmap^55^ was used to covert the coordinates between the two assemblies.

### Identifying transposable elements and re-annotating the pikeperch genome

RepeatModeler v2.0.1 (http://www.repeatmasker.org/RepeatModeler/) was used to identify transposable elements (TE) in the genome assembly. To specifically identify miniature inverted-repeat transposable elements (MITE), the open source software MITE-Tracker^56^ was applied. Subsequently, the outputs from RepeatModeler and MITE-Tracker were combined with FishTEDB (http://www.fishtedb.org/) and RepBase^57^, and used as custom repeats library for repeatMasker (http://repeatmasker.org) to classify repetitive elements and estimate their distribution in the enhanced pikeperch genome.

Annotation of gene models was carried out using the funannotate pipeline v.1.5 (https://funannotate.readthedocs.io). First, we obtained transcript and protein sequences of closely related Percidae, including *Perca flavescens*, *Perca fluvialitis*, *Etheostoma spectabile*, and *Etheostoma cragini*. Second, RNA-Seq from different pikeperch tissues (unpublished data) were aligned to the soft-masked genome with HISAT2^58^ followed by StringTie2^59^ to reconstruct transcripts. Further, augustus v3.2^60^, GlimmerHMM^61^, SNAP^62^, and GeneMark^63^ were used for de novo predictions of protein coding genes. Finally, funannotate was used to integrate transcript evidences, proteins- and transcripts alignments, and de novo gene models to create a consensus gene set. The final gene set was obtained by filtering out genes which were either too short (< 50 amino acids), or had in-frame stop-codons or with significant open reading frame (ORF) homology to TE sequences.

In addition, we predicted the three main types of non-coding RNAs, which play important roles in different cellular processes. We applied tRNAscan-SE algorithm^64^ to identify transfer RNA (tRNA) genes in pikeperch genome. The ribosomal RNA (rRNA) including 5S, 18S and 28S were predicted with RNAmmer algorithms v1.2^65^. MicroRNA (miRNA) was predicted using the standalone version of miRNAFold^66^.

Functional annotations of predicted protein-coding genes were carried out based on several functional databases, including Swissprot, Tr-EMBL, NCBI-NR, KEGG, and eggNOG. Association of gene products with Go-terms was performed using Blast2GO within the OmicsBox suite^67^. We also used InterProScan v5^68^ to map protein domains in the InterPro database, which includes CATH-Gene3D, CDD, HAMAP, PANTHER, Pfam, PIRSF, PRINTS, ProDom, PROSITE, SMAT, SUPERFAMILY, and TIGRFAMs. Lastly, high scoring functional annotations in each database were retained as the final consensus functional annotation results.

## Supplementary information

Supplementary Information 1.

Supplementary Information 2.

Supplementary Information 3.

Supplementary Information 4.

## Data Availability

The raw sequencing data used in this project is available at the NCBI Sequence Read Archive (SRA) under Accession Number PRJNA626522. The annotated assembly of *Sander lucioperca* is available at the NCBI GenBank under the Accession Number GCA_008315115.2.
