# Supplementary material for: An ultra-high density SNP-based linkage map for enhancing the pikeperch (Sander lucioperca) genome assembly to chromosome-scale
Source: Sci Rep. 2020 Dec 18;10:22335. doi: 10.1038/s41598-020-79358-z (PMC7749136; doi:10.1038/s41598-020-79358-z)
Supplement: Supplementary file 1 — Supplementary Information 1. [file 41598_2020_79358_MOESM1_ESM.pdf]

An ultra-high density SNP-based linkage map for enhancing the pikeperch (*Sander lucioperca*) genome assembly to chromosome-scale

Lidia de los Ríos-Pérez[1], Julien A. Nguinkal[2], Marieke Verleih[2], Alexander Rebl[2], Ronald M. Brunner[2], Jan Klosa[1], Nadine Schäfer[2], Marcus Stüeken[3], Tom Goldammer[2,4]*, Dörte Wittenburg[1]*

[1]Institute of Genetics and Biometry, Leibniz Institute for Farm Animal Biology (FBN), Wilhelm-Stahl-Allee 2, 18196 Dummerstorf, Germany.
[2]Institute of Genome Biology, Leibniz Institute for Farm Animal Biology (FBN), Wilhelm-Stahl-Allee 2, 18196 Dummerstorf, Germany.
[3]Mecklenburg-Vorpommern Research Centre for Agriculture and Fisheries, Malchower Chaussee 1, 17194 Hohen Wangelin, Germany.
[4]Molecular Biology and Fish Genetics, Faculty of Agriculture and Environmental Sciences, University of Rostock, 18059 Rostock, Germany.

E-mail: wittenburg@fbn-dummerstorf.de (D.W.); tom.goldammer@uni-rostock.de (T.G.)

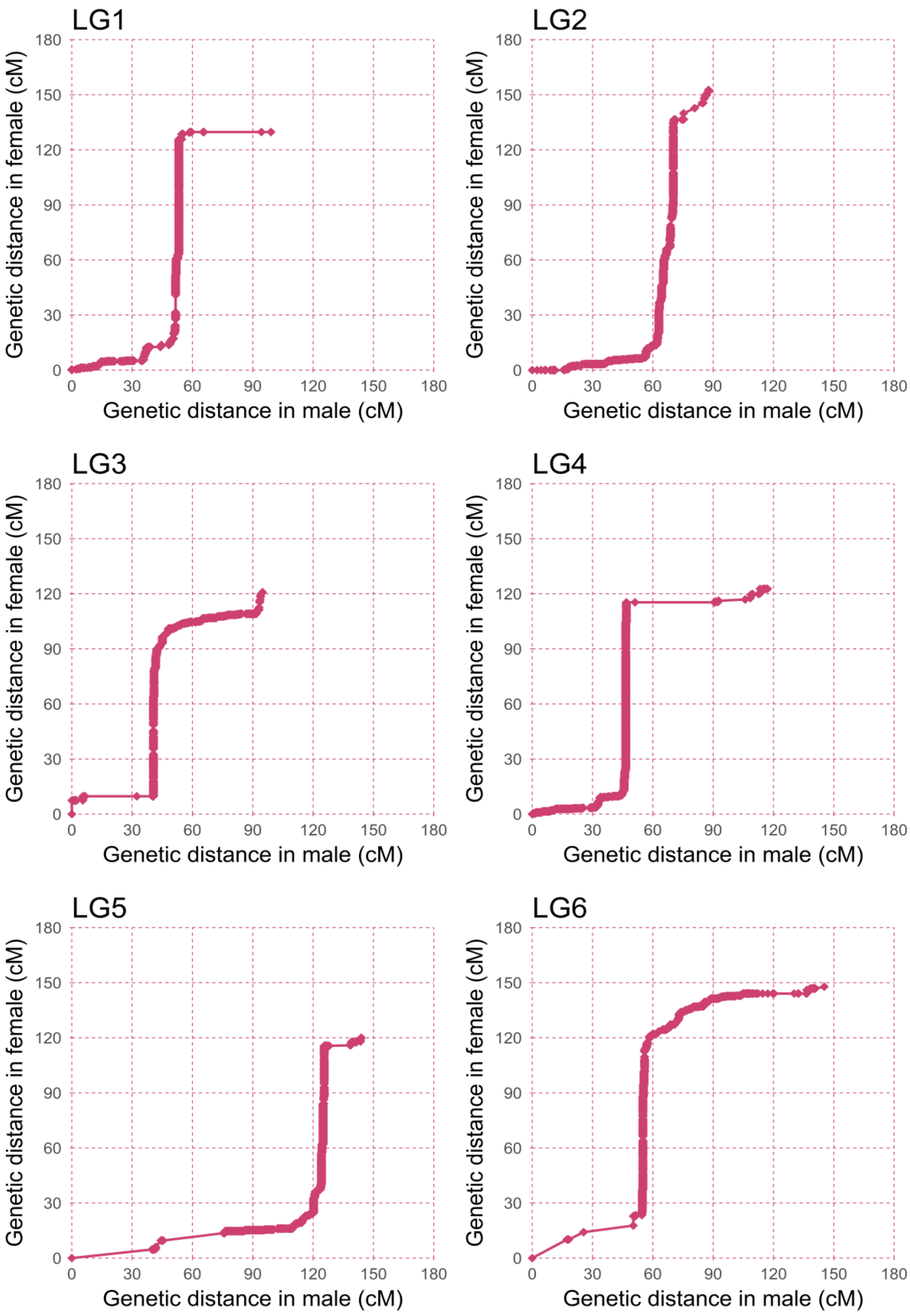
LG1
LG2
LG3
LG4
LG5
LG6
Genetic distance in female (cM)
Genetic distance in male (cM)
0
30
60
90
120
150
180

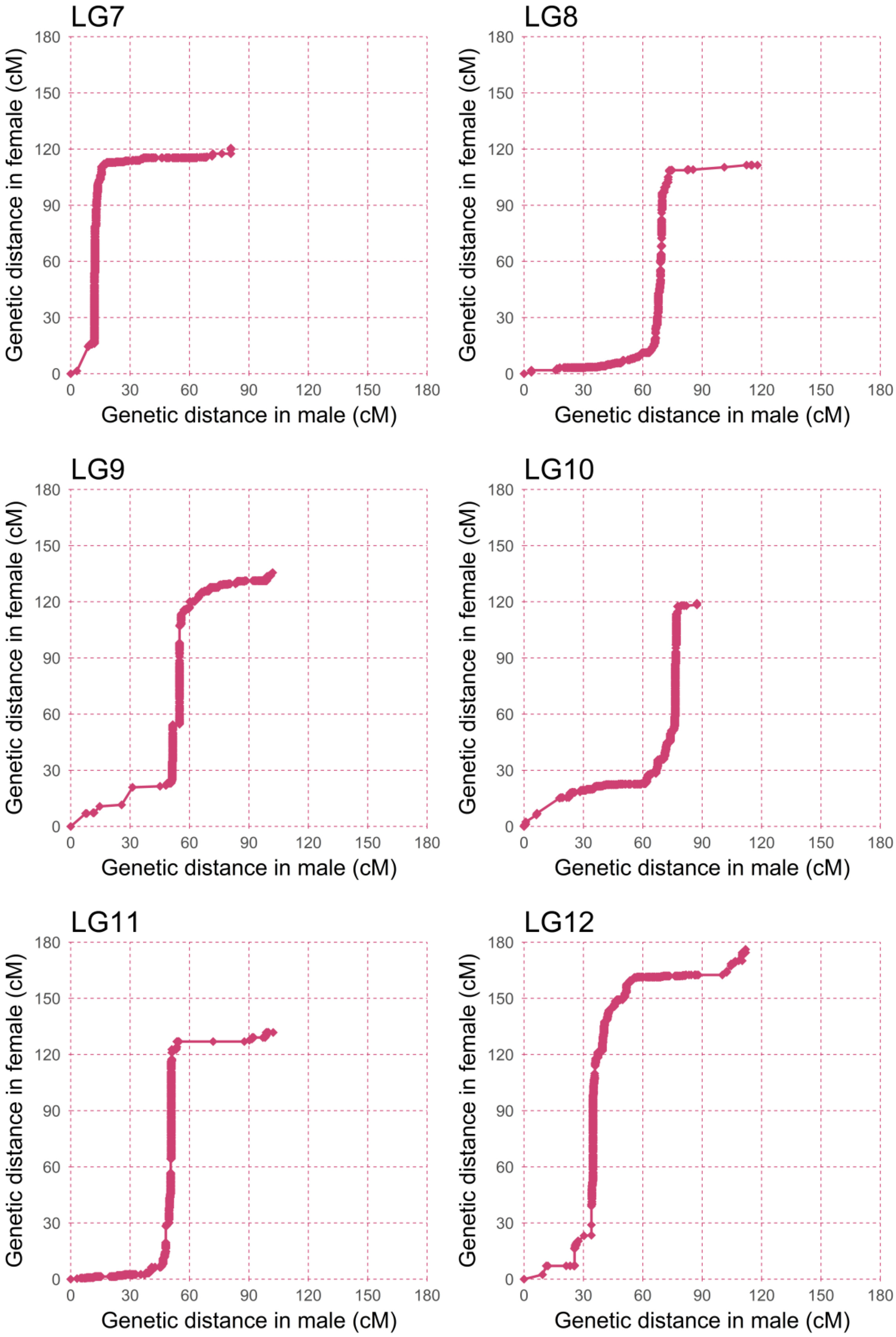
LG7
LG8
LG9
LG10
LG11
LG12
Genetic distance in female (cM)
Genetic distance in male (cM)
0
30
60
90
120
150
180

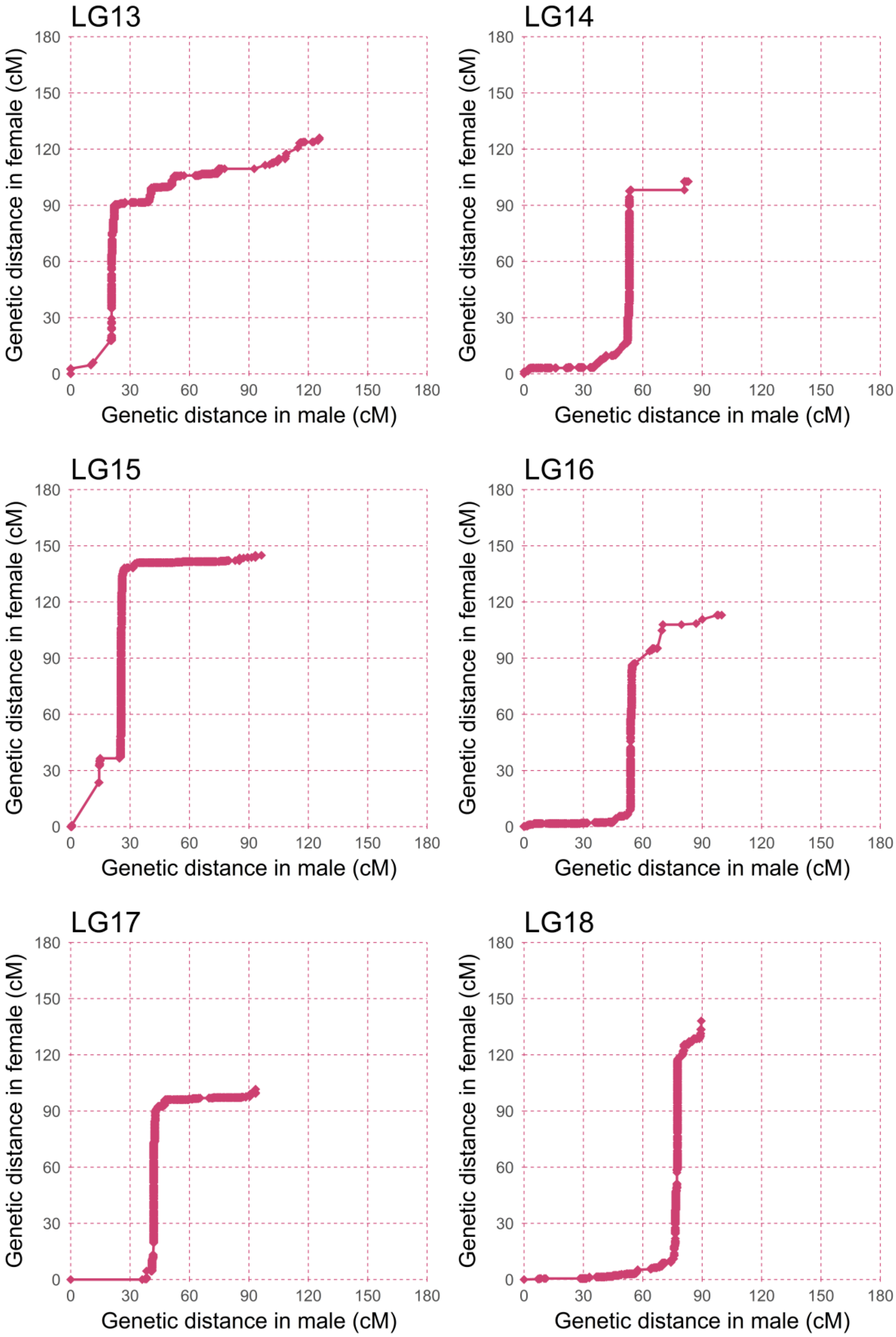
LG13
LG14
LG15
LG16
LG17
LG18
Genetic distance in female (cM)
Genetic distance in male (cM)
0
30
60
90
120
150
180

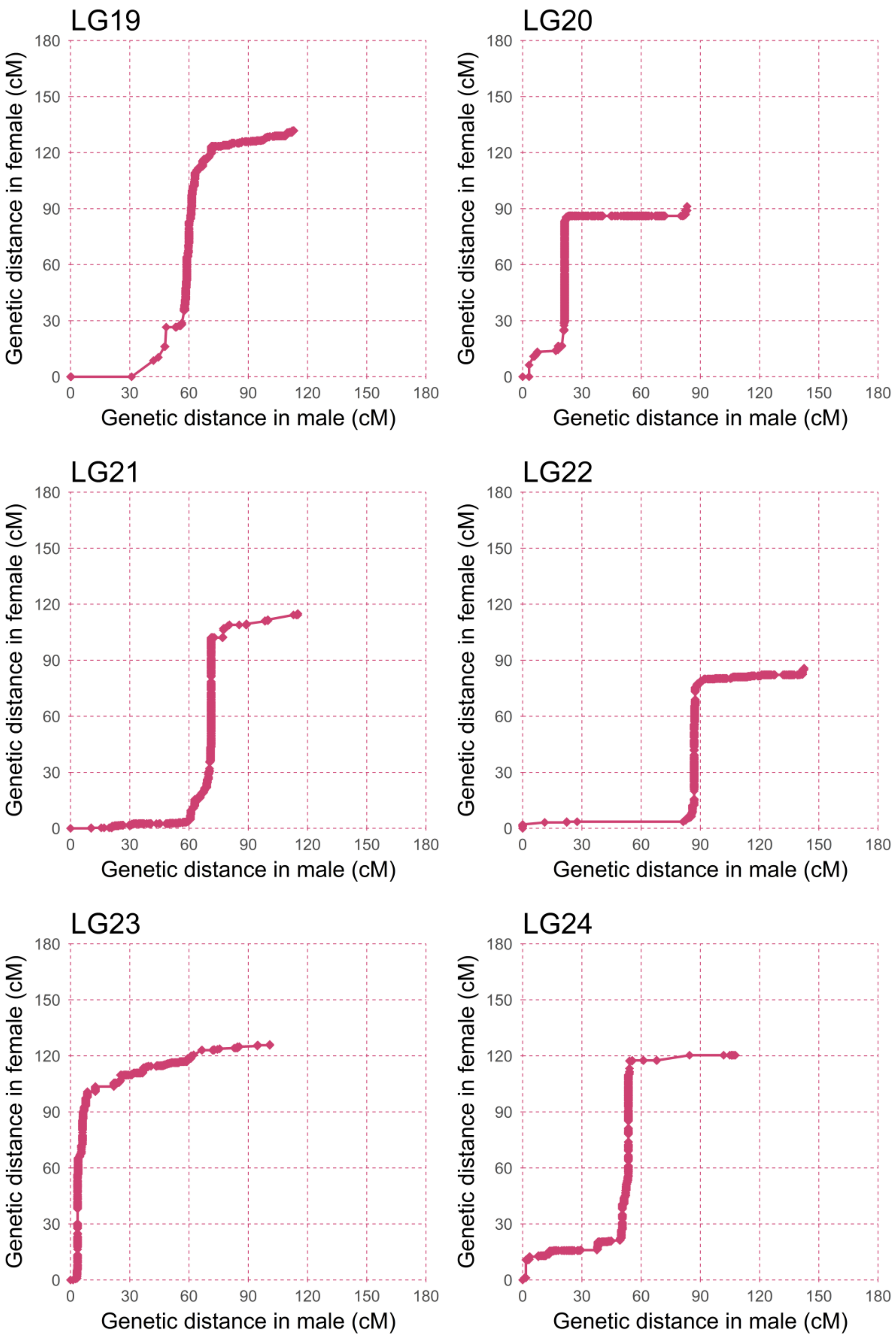

Supplementary Figure S1. Male versus female genetic distances separated by linkage group. The X and Y axis show the cumulative genetic distance in males and females, respectively. Diamonds indicate a change in genetic distance in either male or female.
