# Supplementary material for: An ultra-high density SNP-based linkage map for enhancing the pikeperch (Sander lucioperca) genome assembly to chromosome-scale
Source: Sci Rep. 2020 Dec 18;10:22335. doi: 10.1038/s41598-020-79358-z (PMC7749136; doi:10.1038/s41598-020-79358-z)
Supplement: Supplementary file 2 — Supplementary Information 2. [file 41598_2020_79358_MOESM2_ESM.pdf]

**An ultra-high density SNP-based linkage map for enhancing the pikeperch (*Sander lucioperca*) genome assembly to chromosome-scale**

Lidia de los Ríos-Pérez[1], Julien A. Nguinkal[2], Marieke Verleih[2], Alexander Rebl[2], Ronald M. Brunner[2], Jan Klosa[1], Nadine Schäfer[2], Marcus Stüeken[3], Tom Goldammer[2,4]*, Dörte Wittenburg[1]*

[1]Institute of Genetics and Biometry, Leibniz Institute for Farm Animal Biology (FBN), Wilhelm-Stahl-Allee 2, 18196 Dummerstorf, Germany.
[2]Institute of Genome Biology, Leibniz Institute for Farm Animal Biology (FBN), Wilhelm-Stahl-Allee 2, 18196 Dummerstorf, Germany.
[3]Mecklenburg-Vorpommern Research Centre for Agriculture and Fisheries, Malchower Chaussee 1, 17194 Hohen Wangelin, Germany.
[4]Molecular Biology and Fish Genetics, Faculty of Agriculture and Environmental Sciences, University of Rostock, 18059 Rostock, Germany.

E-mail: wittenburg@fbn-dummerstorf.de (D.W.); tom.goldammer@uni-rostock.de (T.G.)

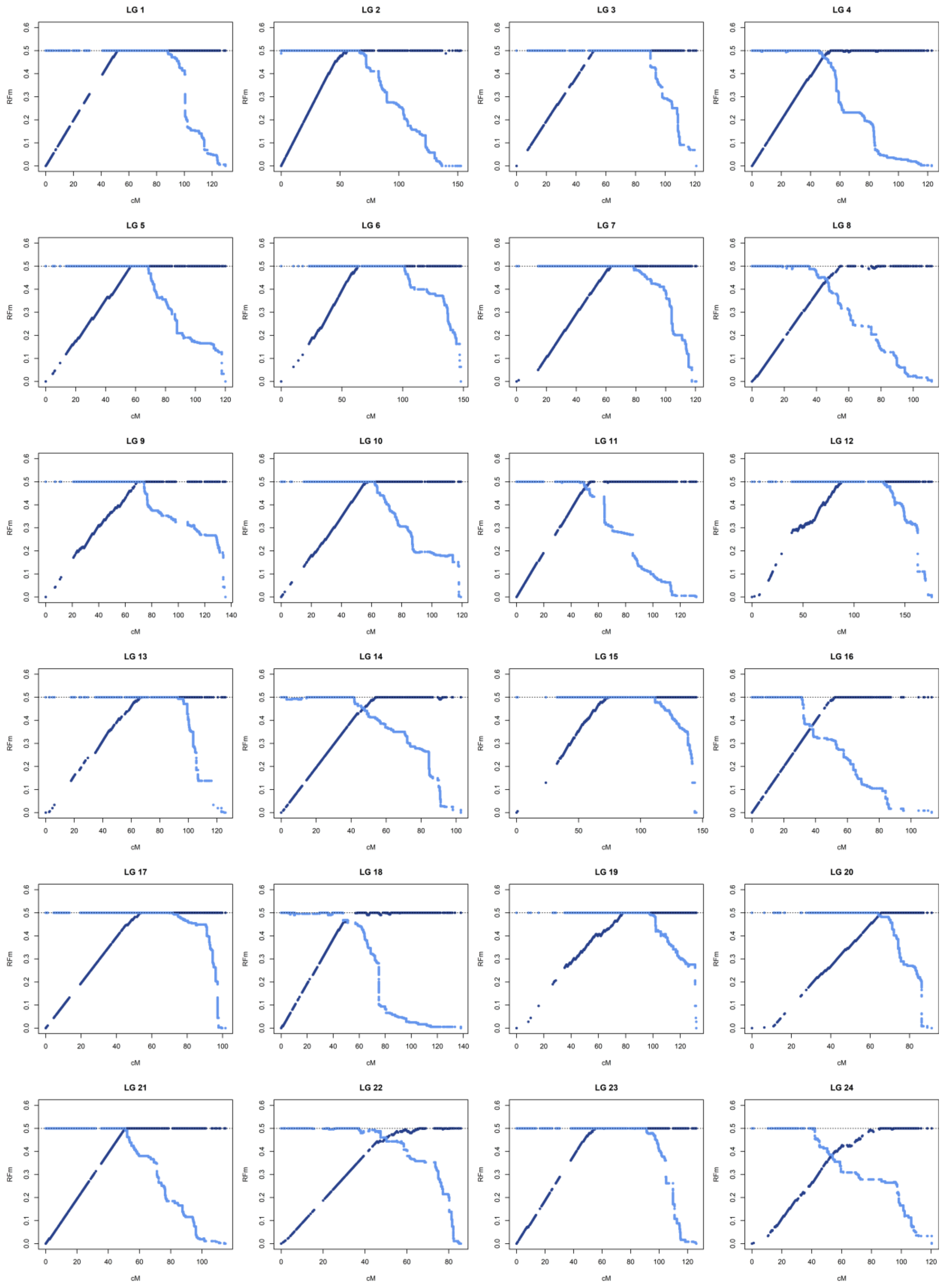


**Supplementary Figure S2.** Recombination frequencies (RF) for intervals between a terminal and any other marker (m) on each linkage group of the female map. Recombination events were counted from the first until the last SNP (cM = 0; dark blue circles) and from the last until the first SNP (light blue circles).
